# Preload and friction in an implant–abutment–screw complex including a carbon-coated titanium alloy abutment screw: an in vitro study

**DOI:** 10.1186/s40729-023-00473-3

**Published:** 2023-03-22

**Authors:** K. Sagheb, C.-I. Görgen, S. Döll, I. Schmidtmann, S. Wentaschek

**Affiliations:** 1 Department of Prosthodontics and Materials Science, University Medical Center of the University of Mainz, Augustusplatz 2, 55131 Mainz, Germany; 2 Institute for Medical Biostatistics, Epidemiology and Informatics, University Medical Center of the University of Mainz, Obere Zahlbacher Straße 69, 55131 Mainz, Germany

**Keywords:** Dental implant, Abutment screw, Preload, Friction

## Abstract

**Purpose:**

An experimental approach was designed to measure the preload force, the coefficient of friction and the component of the tightening torque that is needed to surmount the thread-friction in an implant–abutment–screw complex that includes a carbon-coated screw. With the determined preload values the coefficient of friction was calculated.

**Methods:**

25 unused complexes, containing an implant, an abutment and a carbon-coated titanium alloy abutment screw, were tested. A custom load frame with two load cells and associated electronics was used. The threads were not lubricated. All abutment screws were torqued ten times to 25 Ncm. The produced preload values and a force that was proportional to the thread-friction component of the tightening torque were recorded.

**Results:**

Mean preload values decreased significantly with the number of repetitions (*p* < 0.0001) from initially 329.9 N ± 33.3 (range 255.7 to 383.9) to 253.7 N ± 36.8 (range 200.1 to 332.5) for the last tightening procedure. The corresponding change in the calculated coefficient of friction was 0.33 ± 0.04 (range 0.28 to 0.43) to 0.44 ± 0.07 (range 0.32 to 0.56). For the thread-friction no corresponding trend for consecutive tightening repetitions could be noticed.

**Conclusions:**

In the investigated implant–abutment units, repeated use of a coated abutment screw appears to increase the friction of the screw head and thereby decrease the preload. These results indicate that a pre-used coated implant–abutment–screw will fail reaching optimal screw preload.

**Graphical abstract:**

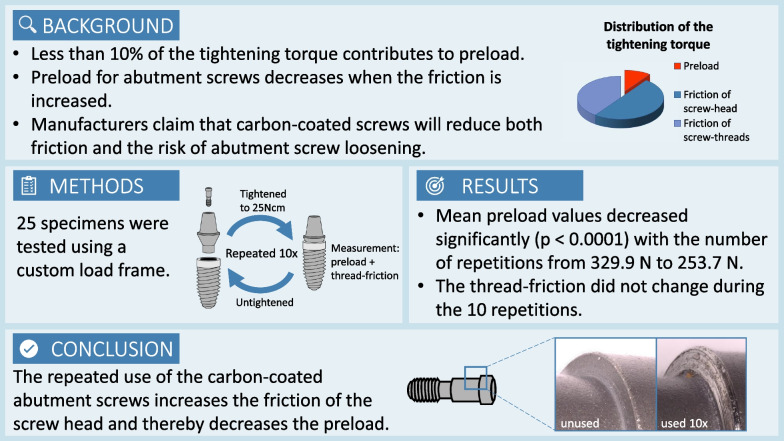

## Background

One of the most common mechanical complications regarding implant superstructures is the loosening of abutment screws, which might occur under masticatory loading [1]. Screw loosening can lead to various problems, like biological complications due to microleakage at the increased gap between the implant and the abutment [2]. In order to avoid biological complications and to maintain a stable peri-implant situation, it is crucial to prevent screw loosening [3]. The force that keeps the parts of a screwed implant–abutment complex with a butt-joint connection together is called preload. Optimal preload decreases the risk of abutment screws loosening and contributes to reduce microleakage [4]. Preload is the mechanical stress that arises when the applied torque causes the screw to stretch. It is a function of the applied torque as well as the friction of the screw head and the screw threads [5]. The friction depends on the material’s properties, the type and presence of lubrication and is mathematically indicated by the coefficient of friction (COF). An increased COF leads to decreased preload values, and conversely, a decreased COF leads to increased preload values using the same tightening torque. In mechanical engineering, it is assumed that it needs approximately 90% of the tightening force to overcome the friction—50% for the friction of the screw head and 40% for the friction of the screw threads. Consequently, only 10% of the tightening force generates the preload. For this reason, some manufacturers offer screws with a treated surface to reduce friction, and thereby achieve a higher preload with the same tightening torque [6].

There is a mathematical relationship between the applied torque and the preload force, but the coefficient of friction must be known to calculate the preload [7]. For standardized screws, tables with coefficient of friction data are published, but they vary widely and are not specific to our particular implant interface combinations.

Because the COF of the coated screws used in the present implant–abutment units was unknown, this in vitro study aimed to evaluate the preload, calculate the COF, and determine the tightening torque needed to overcome the thread-friction when repeatedly tightening–untightening the screw. The null hypothesis was that the repeated tightening sequences do not lead to significant changes in preload.

## Methods

### The load frame device

**Fig. 1:**
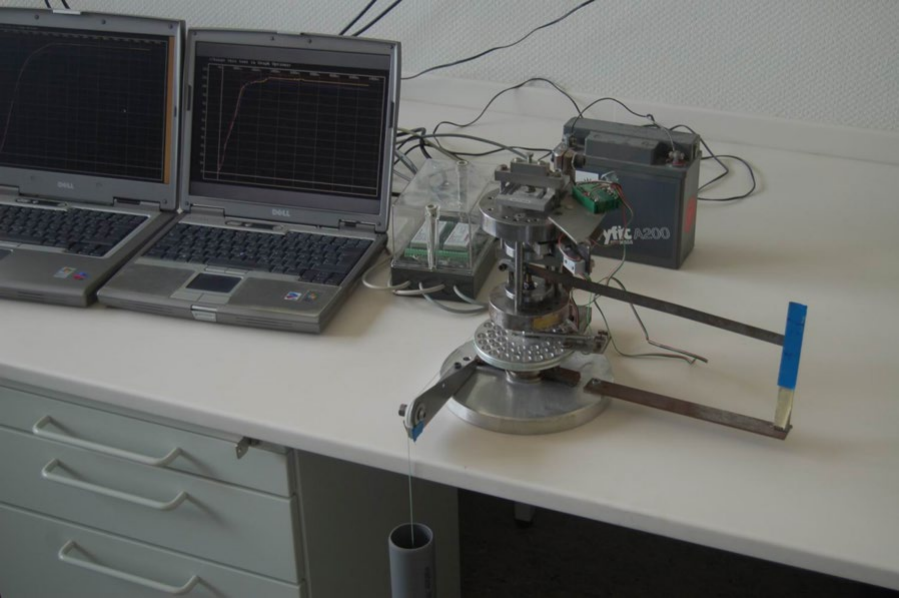
Photography of the measuring station

**Fig. 2:**
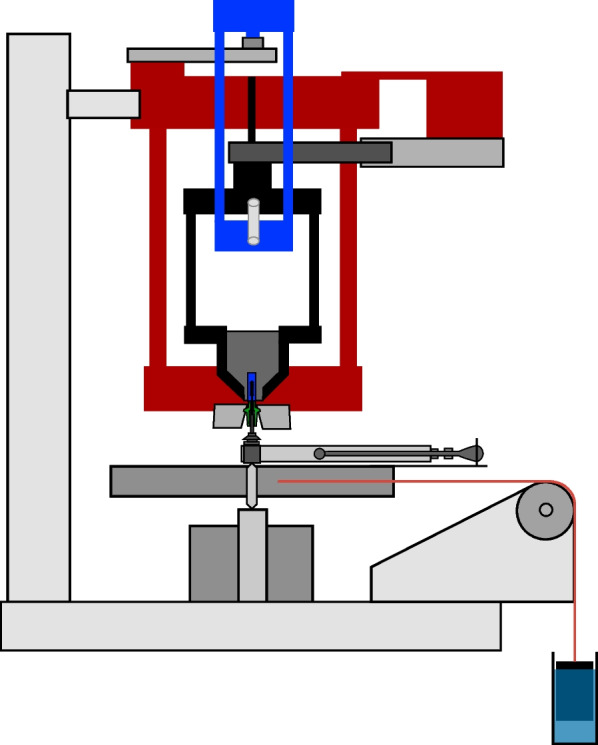
Diagram of the measurement device

A custom load assembly (Figs. 1, 2) was used to measure the tension within the abutment screw when it was tightened. One essential requirement for this measuring method is that the two parts, which are screwed together, must not come in contact with each other. For that, a gap of 0.10 mm between the abutment and the implant was assured at the end of the tightening procedure.

The upper part was constructed to hold and position the implant components and measure the preload as well as the thread-friction component of the tightening torque. With the lower part, the tightening torque was reproducibly generated.

The upper part was composed of an immovable frame with two horizontal plates rigidly held together by three side columns (Fig. 2, red frame). In this frame, a free-rotating second frame with two horizontal plates and three side columns was centrically pivoted (Fig. 2, black frame). The implant was fixed at the lower tip of the second frame. With a third frame, made of two platforms and two side columns (Fig. 2, blue frame), the second frame was mounted on a planar beam load cell (PB-75 kg-C3; Flintec, Meckesheim, Germany), which was located at the top of the device. The load cell had a load range of 750 N, and the measuring path at nominal load was 0.35 mm.

When torque was applied to the implant, the third frame loaded the load cell on the top of the device. The freely rotating second frame with the fixed implant was countered against another load cell (Single Point Load Cell 1002-K-Z, Soemer, Lennestadt, Germany) located at the bottom of the device. This load cell had a load range of 150 N, and the measuring path at nominal load was 0.4 mm. The moment of torque generated by the second frame was proportional to the thread-friction component of the implant–abutment–screw complex and to the force needed to overcome the threads’ friction. The abutment was mounted underneath the immovable frame. In the lower part of the device, the tightening torque was delivered steadily by a weight, which pulled at a disc and hereby made the disc rotate. To secure that the disc exerted accurate and reproducible torque values, the weight was dipped into a water-filled tube, and via a cord, over a pulley, the disc was rotated. During the assembly and the manufacturing, it was ensured that all parts were in line with the central loading axis.

The device’s base was a 20-mm-thick aluminum plate. All other components of the load frame were made of steel (E295, according to EN 10,027-1).

The load cells were connected with two load cell digitizing units (LDU 68.1, Hauch & Bach, Lynge, Denmark). These measuring amplifiers communicated via a RS 422/485 full duplex interface with the computer and the corresponding analyzing software (DOP 2.06, Hauch & Bach, Lynge, Denmark). With this application, the load cell digital amplifier devices were calibrated. The application could monitor values in real-time saving the measured data for a previously set interval and duration (here, 0.022-s intervals, 60-s duration).

### Test protocol

25 unused titanium implant–abutment–screw units were tested, each with an implant (Replace Select Tapered implants, Nobel Biocare AG, Zürich, Switzerland), an abutment (Temporary Abutment Non-engaging, Nobel Biocare AG, Zürich, Switzerland) and a carbon-coated abutment screw (Abutment Screw Nobel Replace, Nobel Biocare AG, Zürich, Switzerland). The tests were conducted under dry conditions without lubrication. The abutment screws were torqued ten times to 25 Ncm, maintaining the torque for 60 s before loosening. The produced preload values and the force proportional to the thread-friction component were recorded.

### Calculation of the coefficient of friction and the thread-friction component

**Fig. 3:**
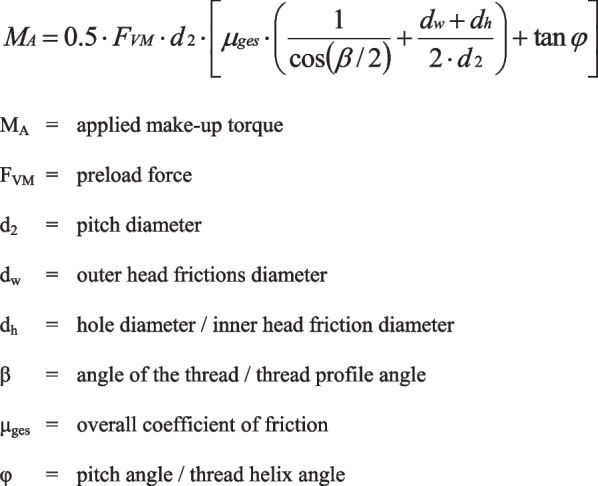
Formula describing the relationship between torque and preload

According to the guideline VDI 2230 Part 1 (Verein Deutscher Ingenieure, Systematic calculation of highly stressed bolted joints—joints with one cylindrical bolt), the mathematic formula (Fig. 3) describing the relationship between the applied torque M_A_ and the preload force F_VM_ incorporates geometric data of the screw connection and the coefficient of friction [5].

**Fig. 4:**
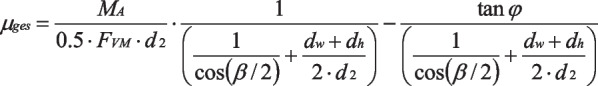
Formula for the coefficient of friction

Since the introduced force was known and the preload was measured, the coefficient of friction could be calculated by solving the before-mentioned formula for it (Fig. 4).

The geometric data of the investigated screw connection were measured in SEM photomicrographs (Zeiss DSM 962 SE, Zeiss, Oberkochen, Germany) with a 50× magnification.

The load cell at the bottom of the device was used to register a force, which was proportional to the thread-friction component of the implant–abutment–screw complex. The measured force could then be converted to the moment of torque, employing the known length of the corresponding lever arm (11 cm).

### Statistical analysis

The statistical software SPSS 23.0 (IBM, Chicago, USA) was used for statistical data analysis. The preload and thread-friction values were analyzed using a linear mixed model with repetition as a fixed effect and the objects as a random effect, thereby taking repeated measurements on each object into account. The significance level was chosen as *α* = 0.05 for each of these two parameters.

When a significant result by the global *F*-test occurred, the Tukey–Kramer test was used for the pairwise evaluation of two repetitions.

## Results

### Preload

**Fig. 5:**
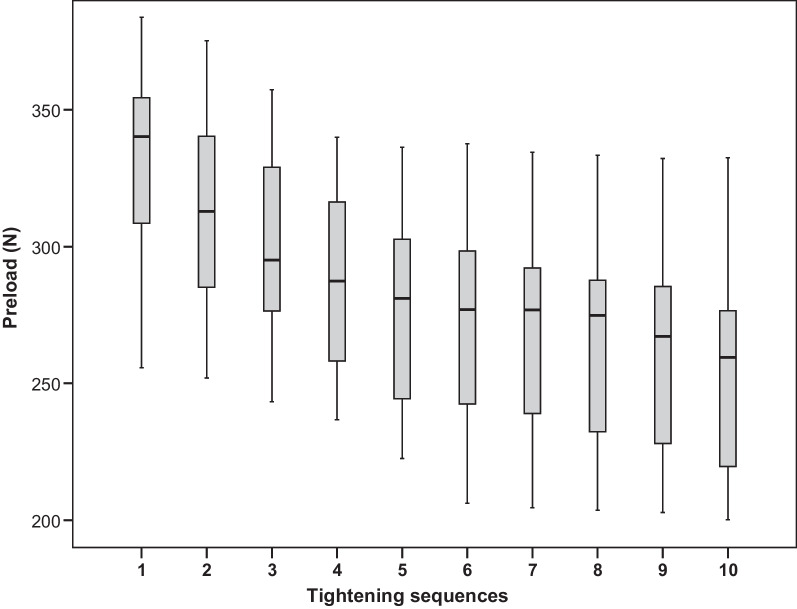
Distribution of preload by tightening sequence

Figure 5 depicts the measured preload values. It shows a noticeable decrease of preload for an increasing number of repetitions (*p* < 0.0001). Overall, for preload, there is a monotone decreasing trend. Table 1 shows the closest repetitions with significantly different preload results for this trend. Preload was significantly lower in the second test than in the first (mean difference = 17.4 N, *p* < 0.0001). In addition, the third test yielded significantly lower preload than the second test (mean difference = 13.8 N, *p* = 0.001). No other preload values were significantly different from the ones directly before.

**Table 1:** First test following any test with significantly different preload

Test no.	First subsequent test with significantly different preload	Difference (*N*)	95% CI (*N*)		Adjusted *p*-value
			Lower B	Upper B	
1	2	17.4	4.1	30.6	0.0016
2	3	13.8	0.6	27.1	0.0328
3	5	19.6	6.4	32.9	0.0002
4	6	14.5	1.3	27.8	0.0192
5	8	14.1	0.8	27.3	0.0270
6	9	15.6	2.3	28.8	0.0082
7	10	16.8	3.5	30.0	0.0029

### Coefficient of friction

The geometric data of the investigated coated screws were needed to calculate the coefficient of friction. These data, which were measured using SEM images, were d_2_ = 1.74 mm, d_w_ = 2.5 mm, d_h_ = 1.99 mm, β = 60° and φ = 4.18°.

**Fig. 6:**
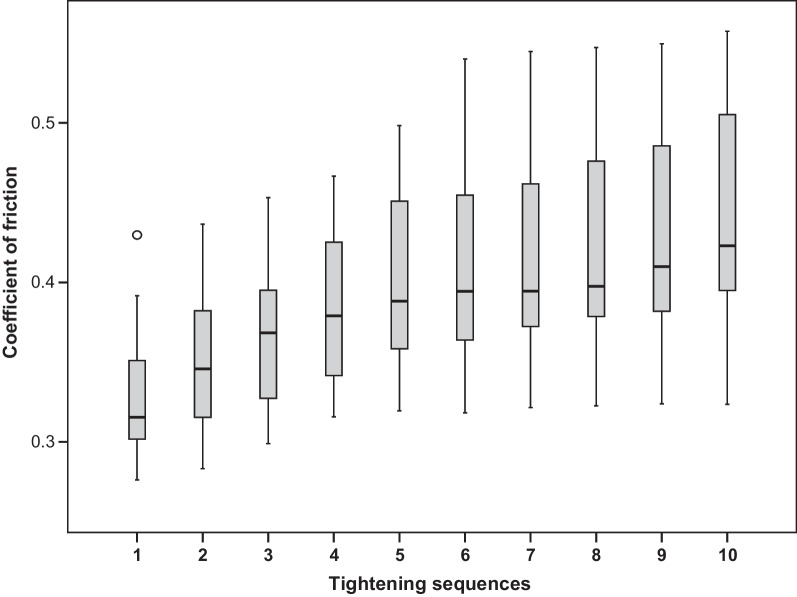
Distribution of the coefficient of friction by tightening sequence

The calculated values of COF are proportional to 1/preload and are presented in Fig. 6.

The friction coefficient is inversely proportional to preload. Consequently, the statistical analysis for COF correlates with the investigation for preload regarding which test repetitions show substantial differences.

### Thread friction component

**Fig. 7:**
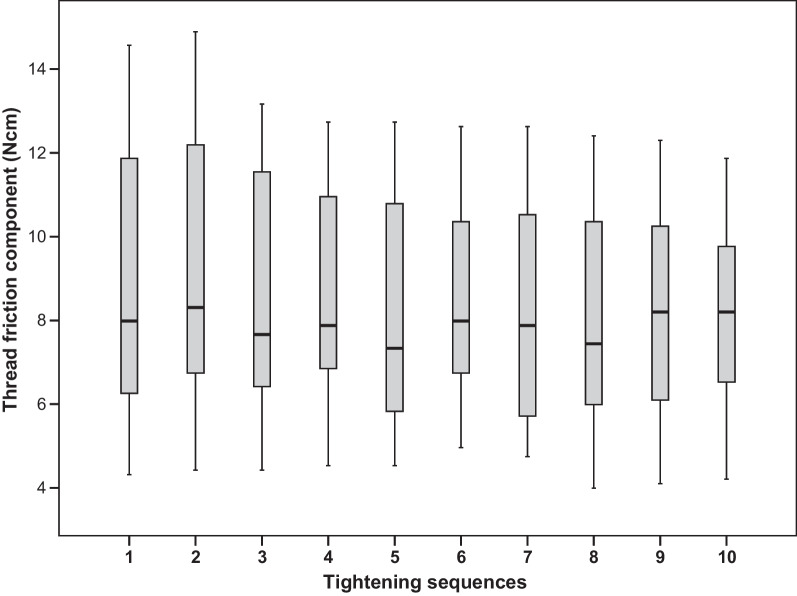
Distribution of the thread-friction component of the tightening torque by tightening sequence

Figure 7 presents the distribution of the thread-friction component of the tightening torque, which was needed to overcome the thread-friction. After 10 consecutive test sequences, no definite trend could be observed for the thread-friction part. Furthermore, there were no significant differences between any mean values (*p* = 0.3005, *F*-test).

## Discussion

With an overall mean coefficient of friction of 0.33, a high friction was found already at the first tightening of the screws. With tightening repetitions, the friction increased due to cumulative wear. Other friction couples commonly used for bolted joints in engineering have much lower coefficients of friction (steel, depending on the lubrication, from 0.08 to 0.16) [5]. But poor friction and wear resistance are known as the main weaknesses of titanium alloys [8], which is why titanium screws fail to reach high preload values [6]. For this reason, some manufacturers offer carbon-coated screws to compensate for this disadvantage by applying a diamond-like carbon surface. In addition, it is reported that when using an equal torque, gold or gold-coated screws could reach higher preload values than titanium screws [9]. In some cases, coated gold screws show even higher preload than coated titanium screws [10, 11]. Other studies found that using different material types for the abutment or for the hybrid abutment–crown has no significant influence on preload [12, 13].

The calculated coefficient of friction at first tightening corresponds to friction coefficients found in other studies, which were made in another context with titanium alloy couples [8, 14].

A high coefficient of friction results in a low preload value. However, our measured preload values differ from some other studies. Martin et al. recorded removal torque values of abutment screws, tightened with 20 and 32 Ncm. They used these removal torque values to calculate preload for abutment screws with treaded surfaces and untreated surfaces. But for comparable titanium alloy abutment screws with treated surfaces and a lower tightening torque than those used in this study, they calculated higher preload values (355.9 N – 470.2 N) [11]. Haack et al. calculated preload after measuring the elongation of titanium screws after tightening with a torque of 20 Ncm, with a preload of 381.5 N, they also calculated higher preload values [15]. However, our results are comparable with the results of Park et al. After tightening different implant–abutment–screw complexes with varying types of connection with a tightening torque of 30 Ncm, they measured preload values of 306–504 N for coated titanium alloy screws depending on the implant type [16]. Zipprich et al. measured preload for self-manufactured components to investigate the influence of the angle of the screw head on preload. When using 25 Ncm tightening torque and screw head angles equal to or larger than 120°, they yielded similar preload results as in this paper [17]. The differing results among papers might be explained due to the diverse methods used to calculate or measure the preload [18].

This study finds that the preload values vary widely within different specimens already at the first tightening test (min. 255.7 N max. 383.9 N), which corresponds to other studies [4, 11, 18, 19]. In engineering, variations in friction are random phenomena with an equally high probability for deviations above or below the mean value [20]. Less than 10% of the tightening torque contributes to the tightening of the screw, and therefore, more than 90% is lost due to friction.  Taking this into account, one could deduce that even small friction differences—caused by small variations in the microstructure or by macroscopically inconspicuous manufacturing defects—could be responsible for the variations in preload [18, 21].

The global F-test showed that mean preload values decreased significantly with the number of repetitions. Therefore, the null hypothesis was rejected. The changes in preload with repeated screw tightening are not consistently described in the literature. Some authors have found an increase in preload with repeated tightening [4, 15, 22], others observed a decrease after retightening [18, 23–25], and in some studies, the preload did not change [9, 10]. These reports of differing preload changes after repeated tightening might be attributed to varying measuring methods, and especially the use of differing materials, which may have individual friction characteristics and wear phenomena. These are the same factors which might cause varying preload values within the first tightening test.

The decrease of the preload for repeated tightening is associated with the increase of overall COF resulting from both thread-friction and head-friction of the screw. However, our measurements showed that thread-friction did not change. Therefore, the increase of COF must have occurred in the screw head. Corresponding surface alterations at the screw head, in the area of the junction between the screw head and the screw shank, were indeed macroscopically visible for all screws after ten tightening tests.

**Fig. 8:**
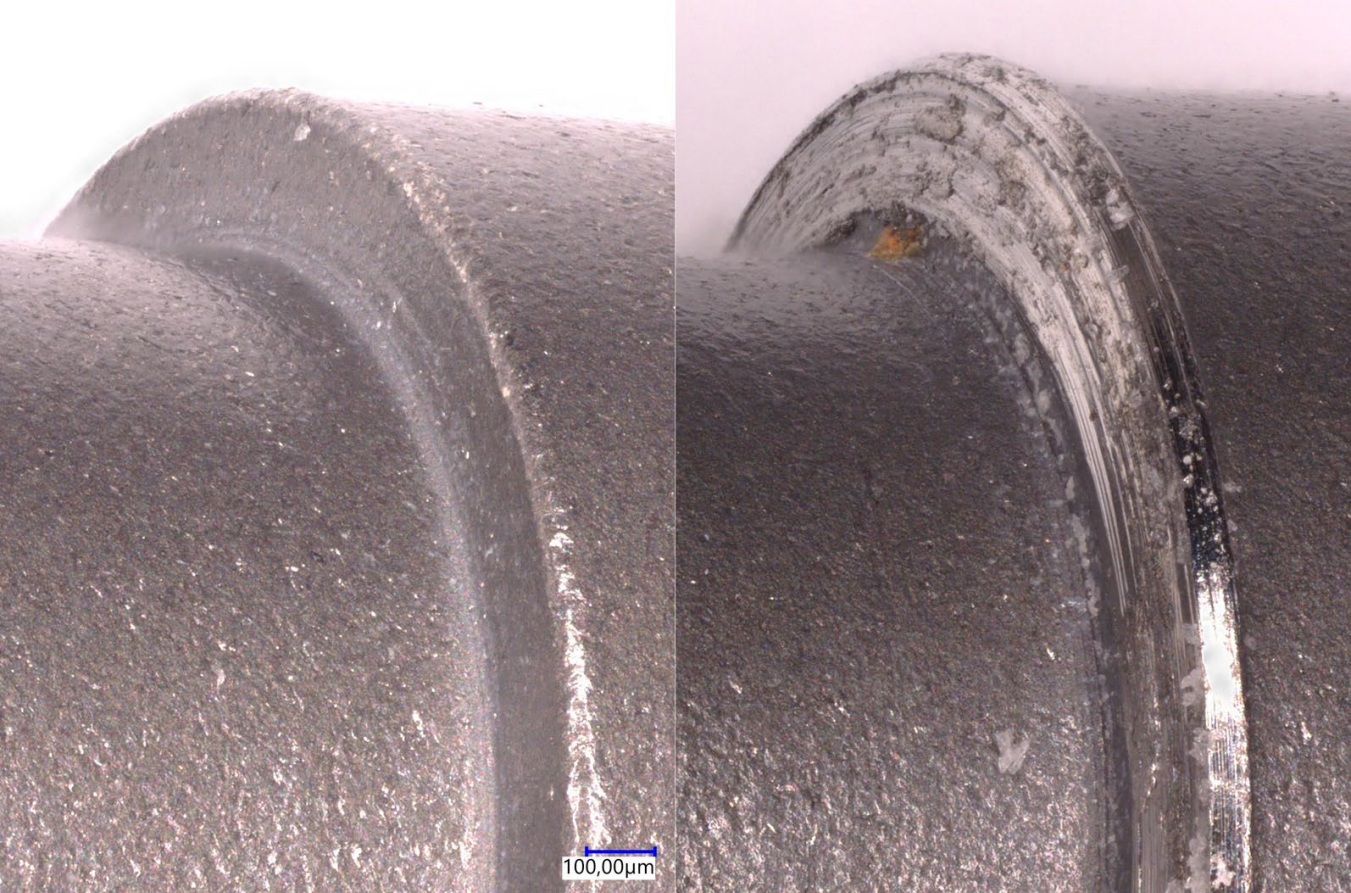
SEM photomicrograph (50x) of the bearing surface of the screw head before (left picture) and after (right picture) ten tightening sequences

Figure 8 shows the effect of repeated tightening on the bearing surface of the screw head in a SEM photomicrograph with a 50x magnification. The left picture in Fig. 8 shows the initially unaffected bearing surface of an unused screw. However, after ten tightening sequences various surface abrasions are visible on the bearing surface for the same screw (right picture in Fig. 8).

These abrasions in the area of the screw head seem to increase the friction and thus decrease the preload. These characteristic wearing effects may occur due to the individual shapes of the bearing of the screw head and the corresponding contact area of the abutment. Therefore, the results might be divergent in other screw connections or in the same connection with another abutment.

The COF values measured at the first tightening sequence in this study were lower than those in a preceding study, where uncoated screws were used, conducting the same measuring procedure. The loss of preload due to repeated tightening was comparable in both studies. As expected, the coated screws achieved higher preload with the application of the same tightening torque [26], but this difference was more pronounced than expected. A prominent similarity in both studies were the macroscopically visible surface alterations at the screw heads after repeated tightening.

A potential problem of low preload values, even at first tightening, is the seal performance of butt-joint connections [27, 28]. Some studies stated that screw retained joints in butt-joint connections may show an increased gap formation even when following the manufacturer’s recommendations for the applied torque strictly [27–29]. By comparison, conical connections are more resistant to abutment movement and microgap enlargement [6, 28, 30]. Independent of the abutment–implant connection type, a sufficiently high preload is crucial for maintaining stability and preventing microgaps [31]. In the synopsis, tightening torques recommended by manufacturers might be too low in some cases, and higher torques would be necessary to mitigate the risk of screw loosening.

## Conclusions

Preload values reached at a given tightening torque can vary widely, and the repeated use of implant–abutment screws could cause decreased preload despite constant tightening torque. These results suggest applying a used screw from the try-in appointment may be unfavorable for obtaining optimal preload.

## Data Availability

All data generated or analyzed during this study are included in this published article [and its Additional files].

## Abbreviations

COF: Coefficient of friction
SEM: Scanning electron microscope
